# 1-Chloro­acetyl-2,6-bis­(2-methoxy­phen­yl)-3,5-dimethyl­piperidin-4-one

**DOI:** 10.1107/S1600536809033674

**Published:** 2009-08-29

**Authors:** G. Aridoss, D. Gayathri, D. Velmurugan, M. S. Kim, Yeon Tae Jeong

**Affiliations:** a Division of Image Science and Information Engineering, Pukyong National University, Busan 608-739, Republic of Korea; b Centre of Advanced Study in Crystallography and Biophysics, University of Madras, Guindy Campus, Chennai 600 025, India

## Abstract

The piperidone ring in the title compound, C_23_H_26_ClNO_4_, adopts a boat conformation with its two out-of-plane C atoms deviating by 0.597 (2) and 0.630 (2) Å from the least-squares plane of the rest of atoms in the ring. The two aromatic rings are roughly perpendicular to each other, making a dihedral angle of 75.1 (1)°, and a C—H⋯π intra­molecular inter­action is observed. The crystal packing is stabilized by a C—H⋯O inter­molecular inter­action, generating a chain with a *C*(9) motif along the *a* axis.

## Related literature

For the biological activity of the piperidine nucleus, see: Weintraub *et al.* (2003 ▶). For the biological activity of piperidones and their *N*-acyl derivatives, see: Perumal *et al.* (2001 ▶); Weintraub *et al.* (2003 ▶); Aridoss *et al.* (2008 ▶, 2009 ▶); Aridoss, Balasubramanian, Parthiban, Ramachandran & Kabilan (2007 ▶). For a related structure, see: Ramachandran *et al.* (2008 ▶). For the synthesis, see: Aridoss, Balasubramanian, Parthiban & Kabilan (2007 ▶). For ring conformational analysis, see: Cremer & Pople (1975 ▶); Nardelli (1983 ▶).
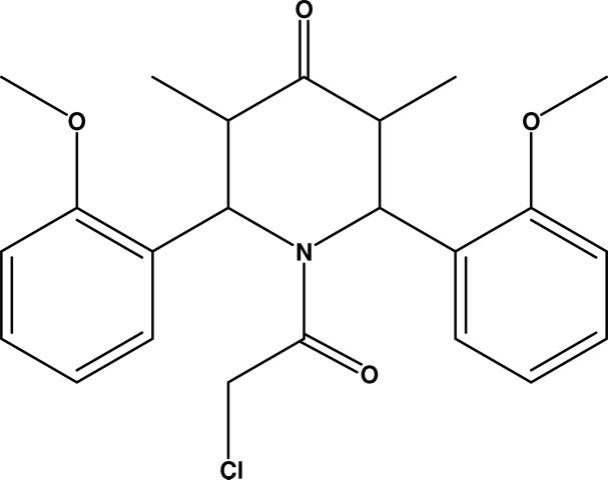


## Experimental

### 

#### Crystal data

C_23_H_26_ClNO_4_
*M* *_r_* = 415.90
Triclinic, 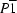

*a* = 8.9921 (6) Å
*b* = 11.3725 (8) Å
*c* = 11.9373 (8) Å
α = 71.630 (1)°
β = 68.465 (1)°
γ = 72.903 (1)°
*V* = 1055.45 (12) Å^3^
*Z* = 2
Mo *K*α radiation
μ = 0.21 mm^−1^
*T* = 293 K
0.27 × 0.22 × 0.20 mm

#### Data collection

Bruker SMART APEX CCD area-detector diffractometer
Absorption correction: none
11114 measured reflections
4603 independent reflections
4054 reflections with *I* > 2σ(*I*)
*R*_int_ = 0.026

#### Refinement

*R*[*F* ^2^ > 2σ(*F* ^2^)] = 0.050
*wR*(*F* ^2^) = 0.145
*S* = 1.05
4603 reflections
266 parameters
H-atom parameters constrained
Δρ_max_ = 0.27 e Å^−3^
Δρ_min_ = −0.35 e Å^−3^

### 

Data collection: *SMART* (Bruker, 2001 ▶); cell refinement: *SAINT* (Bruker, 2001 ▶); data reduction: *SAINT*; program(s) used to solve structure: *SHELXS97* (Sheldrick, 2008 ▶); program(s) used to refine structure: *SHELXL97* (Sheldrick, 2008 ▶); molecular graphics: *PLATON* (Spek, 2009 ▶); software used to prepare material for publication: *SHELXL97* and *PARST* (Nardelli, 1995 ▶).

## Supplementary Material

Crystal structure: contains datablocks I, global. DOI: 10.1107/S1600536809033674/is2452sup1.cif

Structure factors: contains datablocks I. DOI: 10.1107/S1600536809033674/is2452Isup2.hkl

Additional supplementary materials:  crystallographic information; 3D view; checkCIF report

## Figures and Tables

**Table 1:** Hydrogen-bond geometry (Å, °)

*D*—H⋯*A*	*D*—H	H⋯*A*	*D*⋯*A*	*D*—H⋯*A*
C23—H23*C*⋯O1^i^	0.96	2.50	3.321 (3)	144
C9—H9⋯*Cg*	0.93	2.69	3.621 (2)	177

Symmetry code: (i) 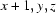. *Cg* is the centroid of the C17–C22 ring.

**Fig. 1.**
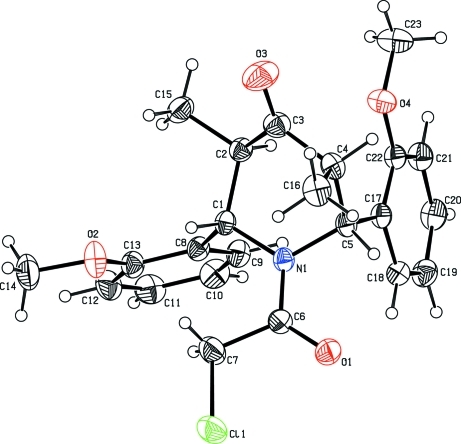
The molecular structure of title compound, (I), showing 30% probability displacement ellipsoids.

**Fig. 2.**
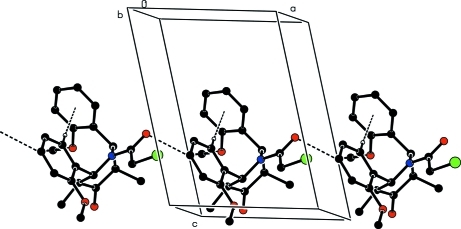
The molecular packing of (I), showing intramolecular C—H···π and intermolecular C—H···O interactions. For clarity, hydrogen atoms which are not involved in hydrogen bonding are omitted.

## supplementary crystallographic information

### Comment

Nitrogen containing heterocycles possess important biological profiles. For instance, the piperidine sub-structure is ubiquitous structural feature of many alkaloids, natural products and drug candidates (Weintraub *et al.*, 2003). During the last decade, there were thousands of piperidine compounds mentioned in clinical and preclinical studies. As well owing to the relevance of piperidone-containing bioactive compounds, synthesis of piperidone derivatives have also attracted considerable attention among chemists and biologist (Perumal *et al.*, 2001). It has been recognized well by our earlier studies that *N*-chloroacetyl and the further functionalized derivatives of 2,6-diarylpiperidin-4-one showed moderate to good antimicrobial, analgesic, antipyretic and antimycobacterial activities (Aridoss, Balasubramanian, Parthiban, Ramachandran & Kabilan, 2007; Aridoss *et al.*, 2008,2009). As an extension to our studies on stereochemistry (Aridoss, Balasubramanian, Parthiban & Kabilan, 2007) and crystal studies (Ramachandran *et al.*, 2008), we report here the X-ray crystallographic study of 1-chloroacetyl-2,6-bis (2-methoxyphenyl)-3,5-dimethylpiperidin-4-one.

The molecular structure of the title compound (I) is shown in Fig.1. The sum of the angles at N1 (358.9 (3)°) is in accordance with *sp*^2^ hybridization. The two phenyl rings in the title compound (I) are nearly perpendicular with the dihedral angle being 75.1 (1)°. Methoxy group attached to the phenyl rings is planar as evident from the torsion angle [(C14—O2—C13—C12 (-3.7 (3)°) and C23—O4—C22—C21 (-1.4 (2)°)]. The torsion angle around O1—C6—C7—Cl1 [0.0 (2)°] indicates the planarity of chloroacetyl moiety. The piperidone ring in the title compound (I) adopts boat conformation with atoms C2 and C5 deviating by -0.597 (2) and -0.630 (2) Å, respectively, from the least-squares plane defined by rest of the atoms (N1/C1/C3/C4) in the ring. The puckering parameters (Cremer & Pople, 1975) and the smallest displacement asymmetry parameters (Nardelli, 1983) for piperidone ring are q_2_ = 0.709 (2) Å, q_3_ = 0.022 (2) Å; Q_T_ = 0.710 (2) Å and θ = 88.3 (1)°.

The molecule of (I) is stabilized by weak C—H···O and C—H···π intramolecular interactions. In C—H···π intramolecular interaction, atom C9 acts as donor to the centroid (*Cg*) of the phenyl ring C17–C22 with H···*Cg* distance of 2.69 Å. The crystal packing is stabilized by C—H···O intermolecular interaction, wherein atom C23 acts as donor to O1 (1 + *x*, *y*, *z*) generating a chain C(9) along the *a* axis.

### Experimental

The title compound was obtained by adopting our earlier method (Aridoss, Balasubramanian, Parthiban & Kabilan, 2007). To a solution of 2,6-bis(2- methoxyphenyl)-3,5-dimethylpiperidin-4-one (1 equiv.) and NEt_3_ (1.5 equiv.) in freshly distilled benzene, chloroacetyl chloride (1 equiv.) in benzene was added in drop wise. Stirring was continued until the completion of reaction. Later, it was poured into water and extracted with ethyl acetate. The combined organic extracts was then washed well with 3% sodium bicarbonate solution, brine and dried over anhydrous sodium sulfate. This upon evaporation and subsequent recrystallization of title compound in distilled ethanol afforded fine white crystals suitable for X-ray diffraction study.

### Refinement

All H atoms were positioned geometrically and refined using a riding model, with C—H = 0.93–0.98 Å, and with *U*_iso_(H) = 1.2*U*_eq_(C) or 1.5*U*_eq_(methyl C).

### Crystal data

**Table:**

C_23_H_26_ClNO_4_	*Z* = 2
*M**_r_* = 415.90	*F*(000) = 440
Triclinic, *P*1	*D*_x_ = 1.309 Mg m^−^^3^
Hall symbol: -P 1	Mo *K*α radiation, λ = 0.71073 Å
*a* = 8.9921 (6) Å	Cell parameters from 3104 reflections
*b* = 11.3725 (8) Å	θ = 1.9–28.0°
*c* = 11.9373 (8) Å	µ = 0.21 mm^−^^1^
α = 71.630 (1)°	*T* = 293 K
β = 68.465 (1)°	Block, colourless
γ = 72.903 (1)°	0.27 × 0.22 × 0.20 mm
*V* = 1055.45 (12) Å^3^	

### Data collection

**Table:**

Bruker SMART APEX CCD area-detector diffractometer	4054 reflections with *I* > 2σ(*I*)
Radiation source: fine-focus sealed tube	*R*_int_ = 0.026
graphite	θ_max_ = 28.0°, θ_min_ = 1.9°
ω scans	*h* = −11→11
11114 measured reflections	*k* = −15→14
4603 independent reflections	*l* = −15→15

### Refinement

**Table:**

Refinement on *F*^2^	Primary atom site location: structure-invariant direct methods
Least-squares matrix: full	Secondary atom site location: difference Fourier map
*R*[*F*^2^ > 2σ(*F*^2^)] = 0.050	Hydrogen site location: inferred from neighbouring sites
*wR*(*F*^2^) = 0.145	H-atom parameters constrained
*S* = 1.05	*w* = 1/[σ^2^(*F*_o_^2^) + (0.0778*P*)^2^ + 0.248*P*] where *P* = (*F*_o_^2^ + 2*F*_c_^2^)/3
4603 reflections	(Δ/σ)_max_ < 0.001
266 parameters	Δρ_max_ = 0.27 e Å^−^^3^
0 restraints	Δρ_min_ = −0.35 e Å^−^^3^

### Special details

**Table:**

Geometry. All e.s.d.'s (except the e.s.d. in the dihedral angle between two l.s. planes) are estimated using the full covariance matrix. The cell e.s.d.'s are taken into account individually in the estimation of e.s.d.'s in distances, angles and torsion angles; correlations between e.s.d.'s in cell parameters are only used when they are defined by crystal symmetry. An approximate (isotropic) treatment of cell e.s.d.'s is used for estimating e.s.d.'s involving l.s. planes.
Refinement. Refinement of *F*^2^ against ALL reflections. The weighted *R*-factor *wR* and goodness of fit *S* are based on *F*^2^, conventional *R*-factors *R* are based on *F*, with *F* set to zero for negative *F*^2^. The threshold expression of *F*^2^ > σ(*F*^2^) is used only for calculating *R*-factors(gt) *etc*. and is not relevant to the choice of reflections for refinement. *R*-factors based on *F*^2^ are statistically about twice as large as those based on *F*, and *R*- factors based on ALL data will be even larger.

### Fractional atomic coordinates and isotropic or equivalent isotropic displacement parameters (Å^2^)

**Table:**

	*x*	*y*	*z*	*U*_iso_*/*U*_eq_	
C1	0.62492 (17)	0.76769 (12)	0.18450 (12)	0.0365 (3)	
H1	0.5887	0.7813	0.1125	0.044*	
C2	0.75304 (18)	0.84927 (13)	0.14741 (13)	0.0409 (3)	
H2	0.7983	0.8264	0.2164	0.049*	
C3	0.6724 (2)	0.98870 (15)	0.13142 (15)	0.0493 (4)	
C4	0.51873 (19)	1.02296 (13)	0.23286 (14)	0.0435 (3)	
H4	0.5333	1.0870	0.2648	0.052*	
C5	0.47941 (17)	0.90843 (12)	0.34028 (13)	0.0376 (3)	
H5	0.3655	0.9344	0.3879	0.045*	
C6	0.33625 (18)	0.76953 (14)	0.31911 (14)	0.0415 (3)	
C7	0.3312 (2)	0.6876 (2)	0.2420 (2)	0.0677 (5)	
H7A	0.4139	0.6108	0.2494	0.081*	
H7B	0.3572	0.7327	0.1556	0.081*	
C8	0.70453 (17)	0.62802 (13)	0.21844 (13)	0.0391 (3)	
C9	0.7535 (2)	0.57969 (14)	0.32461 (14)	0.0457 (3)	
H9	0.7322	0.6323	0.3769	0.055*	
C10	0.8335 (2)	0.45490 (16)	0.35465 (17)	0.0557 (4)	
H10	0.8653	0.4242	0.4261	0.067*	
C11	0.8647 (2)	0.37779 (16)	0.27738 (19)	0.0609 (5)	
H11	0.9186	0.2942	0.2968	0.073*	
C12	0.8177 (2)	0.42187 (16)	0.17127 (19)	0.0591 (4)	
H12	0.8392	0.3681	0.1201	0.071*	
C13	0.73780 (19)	0.54777 (15)	0.14083 (15)	0.0465 (3)	
C14	0.7241 (3)	0.5234 (2)	−0.0467 (2)	0.0764 (6)	
H14A	0.8404	0.4997	−0.0800	0.115*	
H14B	0.6782	0.5702	−0.1128	0.115*	
H14C	0.6792	0.4486	−0.0045	0.115*	
C15	0.8945 (2)	0.82148 (18)	0.03379 (16)	0.0550 (4)	
H15A	0.8536	0.8374	−0.0345	0.083*	
H15B	0.9488	0.7345	0.0516	0.083*	
H15C	0.9704	0.8751	0.0128	0.083*	
C16	0.3767 (2)	1.08034 (17)	0.17668 (17)	0.0568 (4)	
H16A	0.4010	1.1535	0.1113	0.085*	
H16B	0.2784	1.1045	0.2397	0.085*	
H16C	0.3621	1.0188	0.1440	0.085*	
C17	0.57351 (18)	0.85534 (13)	0.43542 (13)	0.0380 (3)	
C18	0.51132 (19)	0.76226 (14)	0.53789 (13)	0.0431 (3)	
H18	0.4129	0.7432	0.5475	0.052*	
C19	0.5920 (2)	0.69789 (16)	0.62510 (15)	0.0516 (4)	
H19	0.5477	0.6372	0.6930	0.062*	
C20	0.7387 (2)	0.72462 (17)	0.61039 (16)	0.0541 (4)	
H20	0.7966	0.6782	0.6662	0.065*	
C21	0.8006 (2)	0.81951 (16)	0.51375 (15)	0.0491 (4)	
H21	0.8982	0.8386	0.5060	0.059*	
C22	0.71660 (18)	0.88681 (13)	0.42754 (13)	0.0400 (3)	
C23	0.9135 (3)	1.0185 (2)	0.3189 (2)	0.0691 (5)	
H23A	0.9001	1.0448	0.3915	0.104*	
H23B	0.9348	1.0872	0.2475	0.104*	
H23C	1.0034	0.9479	0.3092	0.104*	
N1	0.48009 (14)	0.80640 (11)	0.28696 (11)	0.0365 (3)	
O1	0.21671 (13)	0.80127 (12)	0.40297 (11)	0.0529 (3)	
O2	0.68636 (18)	0.59924 (13)	0.03773 (12)	0.0640 (4)	
O3	0.7251 (2)	1.06760 (13)	0.04061 (15)	0.0806 (5)	
O4	0.76967 (14)	0.98287 (11)	0.33137 (11)	0.0502 (3)	
Cl1	0.14021 (6)	0.64709 (5)	0.28760 (5)	0.06999 (18)	

### Atomic displacement parameters (Å^2^)

**Table:**

	*U*^11^	*U*^22^	*U*^33^	*U*^12^	*U*^13^	*U*^23^
C1	0.0373 (7)	0.0359 (6)	0.0342 (6)	−0.0015 (5)	−0.0113 (5)	−0.0103 (5)
C2	0.0401 (7)	0.0400 (7)	0.0398 (7)	−0.0062 (6)	−0.0100 (6)	−0.0097 (5)
C3	0.0534 (9)	0.0401 (7)	0.0506 (8)	−0.0103 (7)	−0.0156 (7)	−0.0050 (6)
C4	0.0497 (8)	0.0330 (6)	0.0496 (8)	−0.0015 (6)	−0.0221 (7)	−0.0102 (6)
C5	0.0380 (7)	0.0351 (6)	0.0411 (7)	0.0000 (5)	−0.0148 (6)	−0.0141 (5)
C6	0.0367 (7)	0.0406 (7)	0.0468 (7)	−0.0039 (5)	−0.0136 (6)	−0.0123 (6)
C7	0.0475 (9)	0.0809 (13)	0.0896 (14)	−0.0185 (9)	−0.0085 (9)	−0.0493 (11)
C8	0.0366 (7)	0.0360 (7)	0.0405 (7)	−0.0016 (5)	−0.0091 (6)	−0.0116 (5)
C9	0.0485 (8)	0.0411 (7)	0.0434 (7)	−0.0006 (6)	−0.0147 (6)	−0.0111 (6)
C10	0.0561 (10)	0.0452 (8)	0.0555 (9)	−0.0002 (7)	−0.0205 (8)	−0.0028 (7)
C11	0.0588 (10)	0.0362 (8)	0.0769 (12)	0.0015 (7)	−0.0197 (9)	−0.0103 (8)
C12	0.0591 (10)	0.0432 (8)	0.0741 (11)	−0.0007 (7)	−0.0146 (9)	−0.0279 (8)
C13	0.0449 (8)	0.0453 (8)	0.0484 (8)	−0.0033 (6)	−0.0105 (6)	−0.0194 (6)
C14	0.0947 (16)	0.0851 (14)	0.0634 (12)	−0.0154 (12)	−0.0221 (11)	−0.0406 (11)
C15	0.0466 (9)	0.0614 (10)	0.0490 (9)	−0.0118 (7)	−0.0026 (7)	−0.0154 (7)
C16	0.0626 (11)	0.0490 (9)	0.0594 (10)	0.0032 (8)	−0.0331 (9)	−0.0098 (7)
C17	0.0393 (7)	0.0368 (6)	0.0383 (7)	−0.0021 (5)	−0.0119 (6)	−0.0139 (5)
C18	0.0432 (8)	0.0455 (7)	0.0399 (7)	−0.0105 (6)	−0.0098 (6)	−0.0111 (6)
C19	0.0613 (10)	0.0489 (8)	0.0395 (7)	−0.0107 (7)	−0.0135 (7)	−0.0059 (6)
C20	0.0576 (10)	0.0576 (9)	0.0464 (8)	−0.0006 (8)	−0.0244 (7)	−0.0109 (7)
C21	0.0462 (8)	0.0541 (9)	0.0503 (8)	−0.0037 (7)	−0.0206 (7)	−0.0154 (7)
C22	0.0411 (7)	0.0388 (7)	0.0406 (7)	−0.0053 (6)	−0.0115 (6)	−0.0134 (5)
C23	0.0613 (11)	0.0673 (12)	0.0800 (13)	−0.0300 (9)	−0.0246 (10)	0.0006 (10)
N1	0.0355 (6)	0.0358 (5)	0.0386 (6)	−0.0020 (4)	−0.0116 (5)	−0.0134 (4)
O1	0.0377 (6)	0.0646 (7)	0.0567 (7)	−0.0090 (5)	−0.0075 (5)	−0.0240 (6)
O2	0.0806 (9)	0.0622 (7)	0.0563 (7)	0.0056 (7)	−0.0293 (7)	−0.0315 (6)
O3	0.0860 (11)	0.0505 (7)	0.0731 (9)	−0.0167 (7)	−0.0029 (8)	0.0063 (6)
O4	0.0521 (7)	0.0488 (6)	0.0535 (6)	−0.0188 (5)	−0.0211 (5)	−0.0029 (5)
Cl1	0.0555 (3)	0.0834 (4)	0.0871 (4)	−0.0249 (2)	−0.0184 (2)	−0.0350 (3)

### Geometric parameters (Å, °)

**Table:**

C1—N1	1.4896 (17)	C12—C13	1.399 (2)
C1—C8	1.5291 (18)	C12—H12	0.9300
C1—C2	1.542 (2)	C13—O2	1.370 (2)
C1—H1	0.9800	C14—O2	1.414 (2)
C2—C3	1.520 (2)	C14—H14A	0.9600
C2—C15	1.525 (2)	C14—H14B	0.9600
C2—H2	0.9800	C14—H14C	0.9600
C3—O3	1.210 (2)	C15—H15A	0.9600
C3—C4	1.510 (2)	C15—H15B	0.9600
C4—C5	1.533 (2)	C15—H15C	0.9600
C4—C16	1.539 (2)	C16—H16A	0.9600
C4—H4	0.9800	C16—H16B	0.9600
C5—N1	1.4866 (17)	C16—H16C	0.9600
C5—C17	1.5308 (18)	C17—C22	1.397 (2)
C5—H5	0.9800	C17—C18	1.400 (2)
C6—O1	1.2238 (18)	C18—C19	1.381 (2)
C6—N1	1.3598 (19)	C18—H18	0.9300
C6—C7	1.521 (2)	C19—C20	1.377 (3)
C7—Cl1	1.755 (2)	C19—H19	0.9300
C7—H7A	0.9700	C20—C21	1.379 (3)
C7—H7B	0.9700	C20—H20	0.9300
C8—C9	1.390 (2)	C21—C22	1.397 (2)
C8—C13	1.396 (2)	C21—H21	0.9300
C9—C10	1.389 (2)	C22—O4	1.3612 (18)
C9—H9	0.9300	C23—O4	1.410 (2)
C10—C11	1.369 (3)	C23—H23A	0.9600
C10—H10	0.9300	C23—H23B	0.9600
C11—C12	1.379 (3)	C23—H23C	0.9600
C11—H11	0.9300		
			
N1—C1—C8	112.28 (11)	O2—C13—C8	116.34 (13)
N1—C1—C2	110.76 (11)	O2—C13—C12	123.70 (15)
C8—C1—C2	109.21 (11)	C8—C13—C12	119.96 (15)
N1—C1—H1	108.2	O2—C14—H14A	109.5
C8—C1—H1	108.2	O2—C14—H14B	109.5
C2—C1—H1	108.2	H14A—C14—H14B	109.5
C3—C2—C15	112.61 (13)	O2—C14—H14C	109.5
C3—C2—C1	110.45 (12)	H14A—C14—H14C	109.5
C15—C2—C1	111.99 (12)	H14B—C14—H14C	109.5
C3—C2—H2	107.2	C2—C15—H15A	109.5
C15—C2—H2	107.2	C2—C15—H15B	109.5
C1—C2—H2	107.2	H15A—C15—H15B	109.5
O3—C3—C4	121.64 (15)	C2—C15—H15C	109.5
O3—C3—C2	122.00 (16)	H15A—C15—H15C	109.5
C4—C3—C2	116.32 (13)	H15B—C15—H15C	109.5
C3—C4—C5	112.21 (11)	C4—C16—H16A	109.5
C3—C4—C16	108.41 (13)	C4—C16—H16B	109.5
C5—C4—C16	110.13 (14)	H16A—C16—H16B	109.5
C3—C4—H4	108.7	C4—C16—H16C	109.5
C5—C4—H4	108.7	H16A—C16—H16C	109.5
C16—C4—H4	108.7	H16B—C16—H16C	109.5
N1—C5—C17	110.48 (10)	C22—C17—C18	117.60 (13)
N1—C5—C4	107.60 (11)	C22—C17—C5	126.74 (13)
C17—C5—C4	121.40 (12)	C18—C17—C5	115.61 (13)
N1—C5—H5	105.4	C19—C18—C17	121.83 (15)
C17—C5—H5	105.4	C19—C18—H18	119.1
C4—C5—H5	105.4	C17—C18—H18	119.1
O1—C6—N1	122.87 (14)	C20—C19—C18	119.29 (15)
O1—C6—C7	121.45 (14)	C20—C19—H19	120.4
N1—C6—C7	115.66 (13)	C18—C19—H19	120.4
C6—C7—Cl1	112.67 (13)	C19—C20—C21	120.68 (15)
C6—C7—H7A	109.1	C19—C20—H20	119.7
Cl1—C7—H7A	109.1	C21—C20—H20	119.7
C6—C7—H7B	109.1	C20—C21—C22	119.85 (16)
Cl1—C7—H7B	109.1	C20—C21—H21	120.1
H7A—C7—H7B	107.8	C22—C21—H21	120.1
C9—C8—C13	118.37 (13)	O4—C22—C21	122.75 (14)
C9—C8—C1	120.37 (12)	O4—C22—C17	116.74 (13)
C13—C8—C1	121.17 (13)	C21—C22—C17	120.49 (14)
C10—C9—C8	121.73 (15)	O4—C23—H23A	109.5
C10—C9—H9	119.1	O4—C23—H23B	109.5
C8—C9—H9	119.1	H23A—C23—H23B	109.5
C11—C10—C9	118.96 (16)	O4—C23—H23C	109.5
C11—C10—H10	120.5	H23A—C23—H23C	109.5
C9—C10—H10	120.5	H23B—C23—H23C	109.5
C10—C11—C12	121.17 (15)	C6—N1—C5	117.12 (11)
C10—C11—H11	119.4	C6—N1—C1	122.55 (11)
C12—C11—H11	119.4	C5—N1—C1	119.22 (11)
C11—C12—C13	119.81 (16)	C13—O2—C14	118.37 (15)
C11—C12—H12	120.1	C22—O4—C23	119.13 (13)
C13—C12—H12	120.1		
			
N1—C1—C2—C3	−47.55 (15)	C11—C12—C13—C8	−0.6 (3)
C8—C1—C2—C3	−171.71 (12)	N1—C5—C17—C22	116.22 (15)
N1—C1—C2—C15	−173.93 (12)	C4—C5—C17—C22	−11.1 (2)
C8—C1—C2—C15	61.92 (15)	N1—C5—C17—C18	−61.14 (16)
C15—C2—C3—O3	−4.8 (2)	C4—C5—C17—C18	171.55 (12)
C1—C2—C3—O3	−130.78 (19)	C22—C17—C18—C19	−3.6 (2)
C15—C2—C3—C4	172.96 (14)	C5—C17—C18—C19	173.98 (13)
C1—C2—C3—C4	46.93 (18)	C17—C18—C19—C20	−0.8 (2)
O3—C3—C4—C5	−177.97 (18)	C18—C19—C20—C21	3.7 (3)
C2—C3—C4—C5	4.31 (19)	C19—C20—C21—C22	−2.0 (2)
O3—C3—C4—C16	60.2 (2)	C20—C21—C22—O4	178.85 (14)
C2—C3—C4—C16	−117.54 (16)	C20—C21—C22—C17	−2.6 (2)
C3—C4—C5—N1	−52.29 (16)	C18—C17—C22—O4	−176.08 (12)
C16—C4—C5—N1	68.57 (14)	C5—C17—C22—O4	6.6 (2)
C3—C4—C5—C17	76.29 (16)	C18—C17—C22—C21	5.3 (2)
C16—C4—C5—C17	−162.84 (12)	C5—C17—C22—C21	−171.99 (13)
O1—C6—C7—Cl1	0.0 (2)	O1—C6—N1—C5	−12.0 (2)
N1—C6—C7—Cl1	−178.78 (13)	C7—C6—N1—C5	166.80 (14)
N1—C1—C8—C9	−54.71 (18)	O1—C6—N1—C1	−179.81 (13)
C2—C1—C8—C9	68.55 (17)	C7—C6—N1—C1	−1.0 (2)
N1—C1—C8—C13	128.78 (15)	C17—C5—N1—C6	109.92 (14)
C2—C1—C8—C13	−107.96 (16)	C4—C5—N1—C6	−115.50 (13)
C13—C8—C9—C10	−0.1 (2)	C17—C5—N1—C1	−81.81 (14)
C1—C8—C9—C10	−176.73 (15)	C4—C5—N1—C1	52.77 (15)
C8—C9—C10—C11	0.0 (3)	C8—C1—N1—C6	−72.11 (16)
C9—C10—C11—C12	−0.2 (3)	C2—C1—N1—C6	165.51 (12)
C10—C11—C12—C13	0.5 (3)	C8—C1—N1—C5	120.29 (13)
C9—C8—C13—O2	179.59 (15)	C2—C1—N1—C5	−2.09 (15)
C1—C8—C13—O2	−3.8 (2)	C8—C13—O2—C14	177.07 (18)
C9—C8—C13—C12	0.4 (2)	C12—C13—O2—C14	−3.7 (3)
C1—C8—C13—C12	176.96 (15)	C21—C22—O4—C23	−1.4 (2)
C11—C12—C13—O2	−179.72 (18)	C17—C22—O4—C23	−179.98 (15)

### Hydrogen-bond geometry (Å, °)

**Table:**

*D*—H···*A*	*D*—H	H···*A*	*D*···*A*	*D*—H···*A*
C23—H23C···O1^i^	0.96	2.50	3.321 (3)	144
C9—H9···Cg	0.93	2.69	3.621 (2)	177

Symmetry codes: (i) *x*+1, *y*, *z*.

## References

[bb1] Aridoss, G., Amirthaganesan, S., Ashok Kumar, N., Kim, J. T., Lim, K. T., Kabilan, S. & Jeong, Y. T. (2008). *Bioorg. Med. Chem. Lett.* **18**, 6542–6548. 10.1016/j.bmcl.2008.10.045 18952418

[bb2] Aridoss, G., Balasubramanian, S., Parthiban, P. & Kabilan, S. (2007). *Spectrochim. Acta Part A*, **68**, 1153–1163. 10.1016/j.saa.2007.01.013 17468043

[bb3] Aridoss, G., Balasubramanian, S., Parthiban, P., Ramachandran, R. & Kabilan, S. (2007). *Med. Chem. Res.* **16**, 188–204.

[bb4] Aridoss, G., Parthiban, P., Ramachandran, R., Prakash, M., Kabilan, S. & Jeong, Y. T. (2009). *Eur. J. Med. Chem.* **44**, 577–592. 10.1016/j.ejmech.2008.03.031 18485539

[bb5] Bruker (2001). *SMART* and *SAINT* Bruker AXS Inc., Madison, Wisconsin, USA.

[bb6] Cremer, D. & Pople, J. A. (1975). *J. Am. Chem. Soc.* **97**, 1354–1358.

[bb7] Nardelli, M. (1983). *Acta Cryst.* C**39**, 1141–1142.

[bb8] Nardelli, M. (1995). *J. Appl. Cryst.* **28**, 659.

[bb9] Perumal, R. V., Adiraj, M. & Shanmugapandiyan, P. (2001). *Indian Drugs*, **38**, 156–159.

[bb10] Ramachandran, R., Aridoss, G., Velmurugan, D., Kabilan, S. & Jeong, Y. T. (2008). *Acta Cryst.* E**64**, o2009–o2010. 10.1107/S1600536808030213 PMC2959452 21201207

[bb11] Sheldrick, G. M. (2008). *Acta Cryst.* A**64**, 112–122. 10.1107/S0108767307043930 18156677

[bb12] Spek, A. L. (2009). *Acta Cryst.* D**65**, 148–155. 10.1107/S090744490804362X PMC2631630 19171970

[bb13] Weintraub, P. M., Sabol, J. S., Kane, J. M. & Borcherding, D. R. (2003). *Tetrahedron*, **59**, 2953–2989.

